# Does mammogram attendance influence participation in cervical and colorectal cancer screening? A prospective study among 1856 French women

**DOI:** 10.1371/journal.pone.0198939

**Published:** 2018-06-21

**Authors:** Aurélie Bertaut, Julien Coudert, Leila Bengrine, Vincent Dancourt, Christine Binquet, Serge Douvier

**Affiliations:** 1 Methodology and Biostatistics Unit, Centre Georges François Leclerc, Dijon, France; 2 Medical Oncology Unit, Centre Georges François Leclerc, Dijon, France; 3 ADECA 21-58, « Association pour le dépistage des cancers Côte-d’Or », Dijon, France; 4 INSERM U1231-EPICAD Team, Burgundy-Franche Comte University, Dijon, France; 5 INSERM CIC1432, University Hospital, Dijon, France; 6 Department of Gynecologic and Oncologic Surgery, University Hospital, Dijon, France; Leibniz Institute for Prevention Research and Epidemiology BIPS, GERMANY

## Abstract

**Background:**

We aimed to determine participation rates and factors associated with participation in colorectal (fecal occul blood test) and cervical cancer (Pap-smear) screening among a population of women participating in breast cancer screening.

**Methods:**

From August to October 2015, a self-administered questionnaire was sent by post to 2 900 women aged 50–65, living in Côte-d’Or, France, and who were up to date with mammogram screening. Polytomic logistic regression was used to identify correlates of participation in both cervical and colorectal cancer screenings. Participation in all 3 screenings was chosen as the reference.

**Results:**

Study participation rate was 66.3% (n = 1856). Besides being compliant with mammogram, respectively 78.3% and 56.6% of respondents were up to date for cervical and colorectal cancer screenings, while 46.2% were compliant with the 3 screenings. Consultation with a gynecologist in the past year was associated with higher chance of undergoing the 3 screenings or female cancer screenings (p<10–4), when consultation with a GP was associated with higher chance of undergoing the 3 screenings or organized cancer screenings (p<0.05). Unemployment, obesity, age>59 and yearly flu vaccine were associated with a lower involvement in cervical cancer screening. Women from high socio-economic classes were more likely to attend only female cancer screenings (p = 0.009). Finally, a low level of physical activity and tobacco use were associated with higher risk of no additional screening participation (p<10–3 and p = 0.027).

**Conclusions:**

Among women participating in breast screening, colorectal and cervical cancer screening rates could be improved. Including communication about these 2 cancer screenings in the mammogram invitation could be worth to explore.

## Introduction

Cancer is the leading cause of death among women. In 2012, 48,753 new cases of breast cancer, 18,926 colorectal cancers and 3,028 cervical cancers were diagnosed among women in France. These three cancer sites account for 44.4% of cancer deaths each year, despite the existence of screening programs [1–3].

In France, women aged 50–65 years are eligible for breast and cervical cancer screening [female cancer screenings], and also for colorectal cancer screening. Both breast and colorectal cancer, as part of organized cancer screening programs, are covered by national health prevention policies. Costs of the exams are supported by social security system. The Breast cancer screening program target women in the 50–74-year age group, who receive an invitation to have a mammogram free of charge every 2 years. This program has been implemented nationwide since 2004 with a quality policy ensuring a free double reading of the images. Over the period 2015–2016, the participation rate in organized breast cancer screening was around 51%, to which should be added around 10% who participate on their own individual initiative outside the organized program (in particular women younger than 50 with family history of breast cancer) [4]. This mammogram participation rate which has been quite stable since 2008 hides disparities between areas mainly in relation with healthcare access [5–7]. In addition to geographical determinants, socio-demographical determinants of breast cancer screening participation are well known. Many studies have shown that being married [8], moderate or high use of health services, employment and socio economic level are all associated with higher screening participation rates [6] [9–10].

Cervical cancer screening shows similar participation rates, 61% between 2010 and 2014 in France, but it is not organized at a national level [11]. French guidelines recommend performing cervical cancer screening (by means of a Pap smear test) every three years after two normal Pap-smears at one-year intervals. Screening is opportunistic, i.e. during a consultation for other purposes, and proposed mostly by gynecologists and some general practitioners (GPs). Conversely, colorectal cancer screening is much less widely implemented, with only 30.8% of French women performing the fecal occult blood test (FOBT) in 2015–2016 [12]. Colorectal cancer screening was implemented in all of France in 2009 after an experimental phase in 22 French geographical administrative areas, and is now available for men and women aged 50 to 74 years.

Younger age, good health status, participation in mammogram screening and regular gynecological follow-up are known predictors of a higher cervical cancer screening participation [9] [13–16]. Regarding colorectal cancer screening, factors shown to be associated with increased participation include younger age, having complementary health insurance, non-smoking status, and participation in other screening programs [17–21]. Most studies that focused on the determinants of participation in colorectal or cervical cancer screening mixed two kinds of populations, namely women who already have a general prevention attitude and participate in breast cancer screening; and women who do not participate. Little is known about colorectal and cervical cancer screening behavior among women who already performed mammogram. However, a better knowledge of these women supposed to feel concerned about their health, would allow reaching specific factors that can constitute some leverage to increase participation to either colorectal or cervical cancers screenings.

We aimed to determine participation rates and factors associated with participation in both organized colorectal and individual cervical cancer screenings among a population of women participating in organized breast cancer screening.

## Methods

### Population

This cross-sectional study was conducted between June and August 2015 in women aged between 50 and 65 years old, resident in Côte-d’Or and participating in breast screening. Côte-d’Or is a French Department located in Burgundy, with a population of 524 144 inhabitants, of whom 270 930 are women, and nearly 54 000 of those women are aged between 50 and 65. To be eligible for inclusion in the present study, the women had to meet the following criteria: 1) having been invited to participate in organized breast and colorectal cancer screenings between 1 January 2011 and 31 December 2014, and 2) having participated in breast screening.

### Sampling strategy and sample size calculation

The eligible source population, comprising 21,136 women, was obtained from the local screening structure (ADECA) that sends mammograms invitation letters to every woman living in the administrative area. Overall, 2,900 women were randomly selected, from the ADECA database, to participate in the study. The sample size was determined assuming that 50% of women would be up-to-date for both colorectal and cervical cancer screening, with a precision of 2.5% and a response rate of 55%.

### Main outcomes

#### Cervical cancer screening

Patients were considered up to date for cervical cancer screening if they had undergone a Pap- smear test within the previous 3 years. Cervical cancer screening in France was an individual initiative at the time of the study.

#### Colorectal screening

Patients were considered up to date for colorectal cancer screening if they had undergone a FOBT test within the previous 2 years. FOBT was used in France for colorectal screening at the time of the study. The ADECA send an invitation letter and the test is then given by the GP during a specific consultation. FOBTs are the analyzed in private practices and results are systematically transmitted to the ADECA.

### Study procedures

The study was approved by the French Data Protection Authority (Commission Nationale de l’Informatique et des Libertés). A self-administered questionnaire was delivered by post to each participant. A reminder letter was sent one month after the first mailing. Besides clinical and demographical questions (age, height, weight, highest level of diploma, current occupation, complementary health insurance underwriting), women were asked about their family history regarding breast, colorectal and cervical cancer, and their medical follow-up (last consultation with a GP, gynecologist or gastroenterologist, last influenza vaccination). Data about their health behavior (consumption of fruit and vegetables, level of physical activity, consumption of alcohol and smoking status) were also recorded. Data on cervical cancer screening were self-reported, since no academic or governmental organization currently takes a census of the number of women participating in cervical cancer testing. Self-reported data on colorectal cancer screening were cross validated using the ADECA database.

### Statistical analysis

Categorical variables are presented as number (percentage) and continuous variables as mean± standard deviation (SD). Categorical variables were compared using the Chi square test. When appropriate, continuous variable were dichotomized using the median as a cut-off. Polytomic logistic regression was used to identify variables independently associated with adequate cervical and colorectal cancer screening participation. Participation in all 3 screenings was chosen as the reference. Correlations between eligible variables were tested. The following covariates were considered: age, marital status, BMI, social and occupational group (4 classes), family history of breast, colorectal or cervical cancer, influenza vaccination, gynecologist consultation in the past 12 months, physical activity, level of education, health insurance coverage, smoking status, consumption of fruit and vegetables, and finally alcohol consumption. All covariates with a p value less than 0.20 in univariate analyses were entered into the multivariate model. A backward selection procedure was then applied to identify the factors associated with the outcome, with a significance level of 0.15 or less. Analyses were performed on complete data. Some variables were grouped due to the low number of subjects in certain response classes. All analyses were performed using SAS version 9.3 software (SAS Institute Inc., Cary, NC, USA). A p-value less than 0.05 was considered statistically significant.

## Results

### Study sample

Among the 2 900 selected women, 1 856 agreed to participate, giving a participation rate of 66.3%. The mean age of participants was 58.8 (SD = 3.8) years, range 50 to 65 years. Half the population (n = 917) had a family history of cancer, including breast (n = 604, 32.9%), colorectal (n = 327, 17.8%) and cervical (n = 211, 11.4%) cancer. The characteristics of the study population are shown in Table 1.

**Table 1:** Socio-demographic characteristics of the population.

	n	%
**Age**		
n	1851	
mean [STD]	58.8 [3.8]	
median [min-max]	59 [50–65]	
missing	5	
**BMI**		
n	1833	
mean [STD]	25.0 [4.8]	
median [min-max]	24.2 [15.1–45.8]	
missing	21	
**Marital status**		
Alone	461	25,0%
In couple	1382	75,0%
missing	13	
**Diploma**		
Junior high school degree	279	15,8%
Vocational occupation	581	32,8%
High-School diploma	353	19,9%
High-School diploma + 2 years	245	13,8%
≥ University degree or higher	312	17,6%
missing	86	
**Social and occupational group**		
Farmer	23	1,3%
Self-employed, traders	46	2,5%
Senior manager	184	10,0%
Junior manager	131	7,1%
Employee	556	30,3%
Manual worker	42	2,3%
Unemployed	203	11,1%
Retired	650	35,4%
missing	21	
**Supplementary health insurance**		
No	42	2,3%
Yes	1806	97,7%
missing	8	
**Family history of breast cancer**		
No	1176	64,0%
Yes	604	32,9%
Unknown	58	3,2%
missing	18	
**Family history of cervical cancer**		
No	1494	81,1%
Yes	211	11,4%
Unknown	138	7,5%
missing	13	
**Family history of colorectal cancer**		
No	1342	72,9%
Yes	327	17,8%
Unknown	171	9,3%
missing	16	

Our sample reported having a relatively healthy lifestyle, with half the population consuming fruit and vegetables several times per day, and 80% reporting engaging in physical activity at least once a week. More than 85% of women were non-smokers and reported that they consumed alcohol once a week or less (Table 2). Patients with a family history of colorectal cancer had better gastroenterological follow-up (16.9% declared having consulted a gastroenterologist in the previous year vs 9.3% for women without a history of colorectal cancer, p<10–4).

**Table 2:** Health behaviors characteristics of the population.

	n	%
**Alcohol consumption**		
Every days	68	3.7%
Several times a week	240	13.2%
Once a week	458	25.1%
Less often	671	36.8%
Never	387	21.2%
missing	32	
**Tobacco smoking**		
No	1565	85.1%
Yes	275	14.9%
missing	16	
**Fruit consumption**		
Several times per day	914	49.8%
Once a day	628	34.2%
At least once a week	270	14.7%
Never	22	1.2%
missing	22	
**Vegetable consumption**		
Several times per day	909	49.4%
Once a day	747	40.6%
At least once a week	180	9.8%
Never	4	0.2%
missing	16	
**Physical activity practice**		
Every days	350	19.1%
Several times a week	727	39.7%
Once a week	382	20.9%
Less often	239	13.1%
Never	132	7.2%
missing	26	
**Influenza vaccine**		
Each year	315	17.2%
Every second year	40	2.2%
Less often	137	7.5%
Never	1343	73.2%
missing	21	
**GP consultation in the past 12 months**		
No	163	8.9%
Yes	1675	91.1%
missing	18	
**Gynecologist consultation in the past 12 months**		
No	792	43.2%
Yes	1042	56.8%
missing	22	
**Gastroenterologist consultation in the past 12 months**		
No	1638	88.8%
Yes	206	11.2%
missing	12	

### Screening participation rates

The information about screening participation was available for 1749 (94.2%), 1804 (97.2%) and 1720 (92.7%) women for cervical cancer alone, colorectal cancer alone, and both cervical and colorectal cancer, respectively. Participation rates are presented in Table 3.

**Table 3:** Screening participation rates among women still up to date for organized breast screening.

	only breast cancer screening compliance		breast and cervical cancers screening compliance		breast and colorectal cancers screening compliance		3 screenings compliance			Total	
	n	%	n	%	n	%	n	%	p	n	%*
**Overall population**	192	11.2	552	32.1	181	10.5	795	46.2		1720	**100**
**Age**									**<0.001**		
[50–59[	78	9.3	340	40.5	69	8.2	352	42		839	**100**
[59–65]	113	12.9	212	24.1	112	12.7	443	50.3		880	**100**
**BMI**									**<0.001**		
< 25	78	7.9	321	32.8	82	8.4	499	50.9		980	**100**
[25–30[	58	12.2	164	34.6	50	10.6	202	42.6		474	**100**
≥ 30	53	21.3	61	24.5	47	18.9	88	35.3		249	**100**
**Marital status**									**0.043**		
Alone	57	13.3	139	32.6	54	12.6	177	41.5		427	**100**
In couple	132	10.3	411	32	126	9.8	616	47.9		1285	**100**
**Social and occupational group**									**<0.001**		
Employee, manual worker, farmer, junior manager	62	8.8	251	35.4	72	10.2	323	45.6		708	**100**
Senior manager, self-employed, traders	13	6.1	99	46.2	13	6.1	89	41.6		214	**100**
Unemployed	36	19.1	55	29.1	25	13.2	73	38.6		189	**100**
Retired	76	12.8	144	24.3	71	12	302	50.9		593	**100**
**Diploma**									**<0.001**		
Junior high school	33	12.8	59	22.9	46	17.8	120	46.5		258	**100**
Vocational qualification	69	12.9	171	31.9	52	9.7	244	45.5		536	**100**
High-School diploma	33	10.1	108	32.9	30	9.1	157	47.9		328	**100**
High-School diploma + 2 years	18	7.7	91	39.1	17	7.3	107	45.9		233	**100**
University degree or higher	21	7.1	106	35.7	29	9.8	141	47.4		297	**100**
**Family history of breast cancer**									**0.079**		
No	122	11.1	341	31	113	10.3	524	47.6		1100	**100**
Yes	61	11	193	34.8	55	9.9	246	44.3		555	**100**
Unknown	9	16.7	16	29.6	11	20.4	18	33.3		54	**100**
**Family history of cervical cancer**									**0.262**		
No	148	10.6	455	32.7	145	10.4	645	46.3		1393	**100**
Yes	22	11.3	62	31.8	19	9.7	92	47.2		195	**100**
Unknown	22	17.6	32	25.6	16	12.8	55	44		125	**100**
**Family history of colorectal cancer**									**0.022**		
No	140	11.1	389	30.8	135	10.7	598	47.4		1262	**100**
Yes	28	9.6	117	40	23	7.9	124	42.5		292	**100**
Unknown	23	14.7	42	26.9	21	13.5	70	44.9		156	
**Influenza vaccine**									**0.003**		
Never	142	11.4	430	34.5	117	9.4	557	44.7		1246	**100**
Each year or less often	48	10.4	121	26.3	61	13.3	230	50		460	**100**
**GP consultation in the past 12 months**									**0.063**		
No	20	13.2	61	40.4	12	8	58	38.4		151	**100**
Yes	171	11	488	31.3	169	10.9	728	46.8		1556	**100**
**Gynecologist consultation in the past 12 months**									**<0.001**		
No	175	24	159	21.8	152	20.9	242	33.3		728	**100**
Yes	16	1.6	388	39.8	28	2.9	544	55.7		976	**100**
**Gastroenterologist consultation in the past 12 months**									**0.263**		
No	174	11.3	494	32.2	168	10.9	700	45.6		1536	**100**
Yes	17	9.5	56	31.3	13	7.3	93	51.9		179	**100**
**Supplementary health insurance**									**0.041**		
No	6	15.4	11	28.2	9	23.1	13	33.3		39	**100**
Yes	39	11	539	32.1	172	10.3	782	46.6		1532	**100**
**Physical activity practice**									**<0.001**		
Less often or never	59	17.4	112	32.9	47	13.8	122	35.9		340	**100**
Once a week or more	131	9.6	436	32.1	133	9.8	660	48.5		1360	**100**
**Tobacco smoking**									**0.002**		
No	149	10.3	456	31.5	150	10.3	694	47.9		1449	**100**
Yes	42	16.1	94	36.2	30	11.5	94	36.2		260	**100**
**Alcohol**									**0.248**		
Less often or never	121	12.3	318	32.2	110	11.1	438	44.4		987	**100**
Once a week or more	69	9.8	228	32.2	70	9.9	340	48.1		707	**100**
**Fruit and vegetable consumption**									**0.003**		
At least once per week	17	21	29	35.8	8	9.9	27	33.3		81	**100**
Once a day	67	13	175	34	57	11.1	216	41.9		515	**100**
Several times a day	104	9.4	344	31.1	114	10.3	544	49.2		1106	**100**

BMI: Body Mass Index

*percentages should be read in row. For example 46.2% of the whole population participated to all 3 screenings, while 32.1% participated to breast and cervical cancer screenings only, 10.5% to breast and colorectal cancer screenings only and 11.2% to breast cancer screening only.

Considering each cancer independently, more than ¾ of the responders (n = 1369, 78.3%) were up to date for cervical cancer screening and more than half (n = 1021, 56.6%) for colorectal cancer screening. Only 3.1% (n = 54) had never performed a Pap-smear test and 19.1% (n = 345) had never performed a FOBT test. Regarding the combination of all cancer screenings, 795 (46.2%) were up to date for the 3 cancer types, while 192 (11.2%) were up to date for breast cancer screening only, including 21 women who had never undergone either colorectal or cervical cancer tests. Five hundred and fifty two women (32.1%) were up to date for female cancer screenings only, (i.e. breast and cervical cancers) and 181 (10.5%) for organized cancer screenings only, (i.e. breast and colorectal cancers).

The highest rates of all screening participation were observed among women who had consulted a gynecologist in the past 12 months (55.7%), followed by women with normal BMI, retired women (50.9%) and women aged between 59 and 65 (50.3%).

On the contrary, women who underwent neither cervical nor colorectal cancer screening had not consulted a gynecologist in the past year (24.0%), were mostly unemployed (19.1%) and had a low level of physical activity (less than once a week, 17.4%).

The highest rates of female cancer screenings only were observed among senior managers, the self-employed or traders (46.2%), or women aged less than 59 (40.5%), and those who had not consulted a GP in the past year (40.4%).

Participation rates for organized cancer screening only tended to be systematically lower compared to others modalities (i.e. breast and cervical cancers screening, only breast cancer screening and the 3 screenings compliance).

Regular medical follow up was associated with better screening compliance. Among women who had consulted a GP in the past year or who received the flu vaccine each year, respectively 46.8% and 50% attended the 3 screenings, and only 11.0% and 10.4% underwent mammogram alone. Note that women with regular follow up by a GP were 78.1% to perform cervical cancer screening and 57.7% to perform colorectal screening. Women with regular gynecological follow up either attended all 3 screenings (55.7%) or breast and cervical cancer screening only, as stated above. Only 1.6% of them had mammogram alone and 2.9% were not up to date for cervical cancer screening. On the whole, 95.5% of women who visit a gynecologist in the past year were compliant with cervical cancer screening.

### Polytomic regression

Given their low association with the outcome of interest, family history of cervical cancer (p = 0.262), gastroenterologist consultation in the past 12 months (p = 0.263) and alcohol consumption (p = 0.248) were not considered for multivariate analysis.

After adjustment for confounding factors and compared to women attending all 3 screenings, consultation with a gynecologist in the past year was associated with a higher chance of undergoing the 3 screenings or breast and cervical cancer screenings (lower risk of performing only breast screening (OR = 0.05, p<0.001) or breast and colorectal cancer screenings (OR = 0.09, p<0.001)). In the same time, women who had a GP consultation in the past year were more likely to perform the 3 screenings or breast and colorectal screenings (lower risk of performing only breast cancer screening (OR = 0.52, p = 0.044) or breast and cervical cancer screenings (OR = 0.65, p = 0.034). Women who were unemployed and those who suffer from obesity were more likely to attend no additional screening besides breast cancer screening (OR = 2.75, p = 0.004 and OR = 2.84, p<10–3, respectively) or to be compliant with breast plus colorectal cancer screenings (OR = 1.80, p = 0.061, and OR = 2.22, p = 0.004, respectively). This reflects a lower involvement in cervical cancer screening. For their part, women older than 59 and those who got a yearly flu vaccine were less likely to participate in gynecological cancer screenings only (OR = 0.57, p<0.001 and OR = 0.68, p = 0.008, respectively).

On the contrary, senior managers, self-employed, traders and women with a family history of colorectal cancer were more likely to attend only female cancer screenings, compared to all 3 screenings (OR = 1.65, p = 0.009 and OR = 1.48, p = 0.013, respectively). This reflects a lower involvement in organized colorectal cancer screening.

A low level of physical activity (less than once a week) tend to be associated with worse screening habits, i.e. only female cancer screenings participation (OR = 1.33, p = 0.076), only organized screenings participation (OR = 1.47, p = 0.105) or only breast cancer screening participation (OR = 1.88, p<0.001), even if statistical significance is reach only for the last modality. The same trend is observed among women living alone without achieving statistical significance. Finally, tobacco use was associated with higher risk of no additional screening participation besides breast screening (OR = 1.77, p = 0.027). Results are compiled in Table 4.

**Table 4:** Multivariate polytomic regression.

	only breast screening compliance				breast and cervical cancers screening compliance				breast and CR cancers screening compliance			
	OR	95%CI		p	OR	95%CI		p	OR	95%CI		p
**Age**												
[59–65] *vs* [50–59[	1.10	0.61	1.62	0.995	0.57	0.42	0.78	**<10–3**	1.13	0.71	1.82	0.601
**BMI**												
[25–30 [*vs* < 25	1.54	0.98	2.40	0.67	1.28	0.97	1.681	0.263	1.20	0.77	1.87	0.316
≥ 30 *vs* < 25	2.84	1.67	4.77	**<10–3**	1.17	0.79	1.729	0.872	2.22	1.34	3.70	**0.004**
**Marital status**												
Alone *vs* in couple	1.49	0.97	2.29	0.07	1.25	0.95	1.651	0.114	1.49	0.98	2.26	0.062
**Social and occupational group**												
Senior manager, self-employed, shop-keeper vs ref	1.06	0.52	2.18	0.216	1.65	1.16	2.344	**0.009**	0.92	0.46	1.84	0.336
Unemployed *vs* ref	2.75	1.52	5.00	**0.004**	1.11	0.72	1.705	0.758	1.80	0.98	3.31	0.061
Retired *vs* ref	1.60	0.94	2.73	0.657	1.00	0.71	1.422	0.257	1.17	0.700	1.95	0.955
**Family history of colorectal cancer**												
Yes *vs* no	1.12	0.66	1.89	0.717	1.48	1.10	2.007	**0.013**	0.92	0.54	1.59	0.380
Unknown *vs* no	1.54	0.81	2.93	0.261	0.91	0.59	1.414	0.203	1.44	0.77	2.66	0.218
**Influenza vaccine**												
Each year or less often *vs* never	0.75	0.48	1.17	0.204	0.69	0.53	0.910	**0.008**	1.19	0.79	1.78	0.400
**GP consultation in the past 12 months**												
Yes *vs* no	0.52	0.28	0.98	**0.044**	0.65	0.44	0.967	**0.034**	1.02	0.50	2.09	0.962
**Gynecologist consultation in the past 12 months**												
Yes *vs* no	0.05	0.03	0.09	**<10–4**	1.16	0.89	1.497	0.275	0.09	0.05	0.14	**<10–4**
**Physical activity practice**												
Less often or never *vs* once a week or more	1.88	1.20	2.95	**<10–3**	1.33	0.97	1.818	0.076	1.47	0.92	2.33	0.105
**Tobacco smoking**												
Yes *vs* no	1.77	1.07	2.95	**0.027**	1.33	0.94	1.87	0.103	1.37	0.82	2.30	0.234

OR: odds ratio, CR: colorectal, ref = employee, manual worker, farmer, junior manag

Noticed that no impact of the level of education (p = 0.214), or supplementary health insurance (p = 0.621) or a family history of breast cancer (p = 0.409) on screening habits was observed.

## Discussion

We observed high participation rates in cervical cancer screening (78.3%) and colon cancer screening (56.6%) in this population of women compliant with breast cancer screening. Indeed, in 2011–2012, the participation rates in France for all ages are estimated to be 34% for colorectal cancer and 57% for cervical cancer [13–14] [22–23]. For cervical cancer screening, rates are even lower in the age group included in the present study, reaching about 47% among women aged 60–65 [24]. These higher levels of participation among our population may reflect a better health attitude of women still participating in breast cancer screening compared to the general population. This better health attitude is suggested by a lower rate of tobacco and alcohol use, a higher level of physical activity and a healthier diet compared to the general population [25]. Besides, it has previously been shown that participating in any cancer screening increases the participation rate in other cancer screening types [6] [9] [18]. In terms of public health policy this must be highlighted as promoting one screening may increase participation in the others.

Despite satisfying levels of participation, our population seem more aware of female cancer screenings, suggesting that women feel less concerned by colorectal cancer [21] or that they have a better gynecological follow-up. The types of screening tests are also quite different, which can explain the different participation levels. Mammogram is non-invasive and based on radiologic exam performed by a health care provider. On the contrary, the FOBT is proposed by the GP, but remains a self-administered test. Moreover, many people may feel reluctant to perform the test, which involves fecal manipulation [21] [26]. The new faecal immunobiological test (iFOBT), requiring only one stool sample versus six for the previous test, might be more convenient to use, yielding better screening participation [27–28]. In addition, randomized trails reported that sigmoidoscopy screening reduced colorectal cancer incidence suggesting that FOBT may become of marginal interest in the future [29–31]. Anyways, qualitative studies may be useful to provide explanations and insights into the women’s individual perceptions, thus exploring our results in greater depth. Indeed, this study presents the quantitative results of a larger project that includes both quantitative and qualitative approaches. The next step will be to analyze the psycho-social aspects of screening participation in a specific study consisting in semi-structured qualitative interviews. This will enable us to understand why women who have a preventive approach for one type of cancer failed to participate in specific screenings for other types of cancer (is it a financial concern? The nature of the exam? The representation of the disease? Why breast cancer, and not the other screening exams? Why 2 screenings and not all 3).

Regular follow up by a gynecologist was associated with higher chance of being compliant with all screenings or only female screening. As the same regular follow up by a GP was associated with higher chance of being compliant with all screenings or only organized screenings. This confirms the key-role of these health professionals in cancer prevention [18] [32–33]. In the light of these results, we postulate that encouraging gynecologists to promote colorectal cancers screening, and GPs to encourage cervical cancer prevention, may improve screening coverage even further.

Not surprisingly older women were less likely to participate in cervical cancer screening as stated in many studies [34–36]. Unemployed women compliant with mammogram were more likely to performed no additional screening or only colorectal screening. This impact of socio-economic conditions on cervical screening participation is well known [36–40]. Adherence to organized colorectal cancer screening may be explained as it is free of charge in France. This is a strong argument in favor of the implementation of organized cervical cancer screening as planned in 2018 by the French government [41–42]. The same trend of undergoing no additional screening or only colorectal screening is observed among obese women with less clear explanation as the determinants of obesity are complex. However, in France a significant association between overweight and socio economic level exists [43–44]. Senior managers, self-employed, traders and women with a family history of colorectal cancer were more likely to attend female cancer screenings, compared to all 3 screenings. They are then less involved in colorectal cancer screening. A family history of colorectal cancer is often associated with a better gastroenterological follow-up, explaining lower participation in organized colorectal cancer screening, since these women likely undergo regular colonoscopy outside the context of organized colorectal cancer screening [45]. The same explanation may apply to high socio occupational classes which are more prone to benefit from individual screening as suggested by their high level of Pap smear compliance [40]. A low level of physical activity and tobacco use which may reflect unhealthy habits were associated with higher risk of being compliant with no additional screening, as previously highlighted [36] [46].

Our study found no association between educational level and screening compliance when several other studies did [36–39] [47]. This may be related to the characteristics of our population including women still compliant with breast screening. Noticed that our population had quite similar level of education to French women with 19.9% of high-school degree in our sample *versus* 17.0% in France and 31.4% of women with university degree *versus* 28% [48].

This study presents some limitations that deserve to be underlined. One limitation is the use of self-reported data, which could be affected by biases related to the accuracy of data about screening history. This concerns only cervical cancer screening, since data from the ADECA database of confirmed test results were used to identify colorectal participation and should therefore not impact the results. Furthermore, many studies have concluded that self-reporting is fairly accurate, showing good agreement with administrative health data [49–50]. Self-reported data are also subject to "social desirability" response bias. However this might be limited as our questionnaire was anonymous.

To conclude, colorectal and cervical cancer screening participation rates among women already undergoing breast cancer screening are satisfactory, but leave margin for improvement, especially for colorectal cancer. There still is a need to increase public awareness about the benefit of cancer screening. Encouraging gynecologists to promote colorectal cancer screening and GP to promote cervical cancer screening should be considered with a view to increasing participation rates in cancer screening. In the current context of low medical density in France, including midwives in the prevention offer is also an area that worth exploring to broaden the target audience.
